# An invertebrate-specific miRNA targeted the ancient cholinergic neuroendocrine system of oyster

**DOI:** 10.1098/rsob.160059

**Published:** 2016-08-03

**Authors:** Hao Chen, Zhi Zhou, Lingling Wang, Hao Wang, Rui Liu, Huan Zhang, Linsheng Song

**Affiliations:** 1 Key Laboratory of Experimental Marine Biology, Institute of Oceanology, Chinese Academy of Sciences, Qingdao 266071, People's Republic of China; 2 Key Laboratory of Mariculture and Stock enhancement in North China's Sea, Ministry of Agriculture, Dalian Ocean University, Dalian 116023, People's Republic of China; 3 University of Chinese Academy of Sciences, Beijing 100049, People's Republic of China

**Keywords:** miRNA, miR-2 family, choline transporter-like protein, acetylcholine, cholinergic system, immunomodulation

## Abstract

Acetylcholine (ACh) is the main neurotransmitter in the cholinergic neuroendocrine system and plays an indispensable role in modulating diverse immune responses. As important transporters in choline uptake, choline transporter-like proteins (CTLs) can control ACh synthesis and release indirectly in multiple organisms. In this study, cgi-miR-2d, an invertebrate-specific miRNA in oyster *Crassostrea gigas*, is proved to repress the synthesis/release of ACh by targeting CgCTL1 and choline uptake in haemocytes during the early stage of pathogen infection. In short, an opposite expression pattern between CgCTL1 and cgi-miR-2d is observed during *Vibrio splendidus* infection, accompanied by changes in haemolymph ACh. In addition, the expression level of CgCTL1 is found to be significantly repressed after cgi-miR-2d overexpression *in vivo*, while both haemocyte choline and haemolymph ACh are also decreased simultaneously, similar to the finding in CgCTL1 knock-down assay. As a result, the expression of two tumour necrosis factor-like proteins and the bacteriostatic activity of oyster haemocytes are found to be altered significantly by either gain-of-function cgi-miR-2d or knock-down of CgCTL1. To our knowledge, this is the first miRNA identified in invertebrates that can target the ancient cholinergic system and augment immune response during infection.

## 1. Introduction

The cholinergic system is a pivotal part of the peripheral nervous system and is mainly composed of organized neurocytes that use the neurotransmitter acetylcholine (ACh) in transduction of action potentials [1]. It is reported that the cholinergic system could modulate multiple cognitive functions, such as excitability, learning, memory as well as emotion processing [2–4]. Studies have also suggested the immunomodulatory role of the cholinergic system, which is essential in maintaining the homeostasis of the immune system [5–7]. For example, the expression of tumour necrosis factors (TNFs) and interleukins could be vigorously modulated by increased ACh in serum during lipopolysaccharide (LPS) challenge, contributing to the well-controlled inflammatory response of the host [8,9].

Choline transporter-like proteins (CTLs) are an important class of choline transporters responsible for choline uptake and are expressed in neuronal and some non-neuronal cells [10,11]. The CTLs have been identified in diverse species to date, including vertebrates and invertebrates, and could regulate the synthesis and release of ACh indirectly [12]. Expression of some immune-related genes could be also affected by CTLs [13]. A good balance of CTL expression is therefore crucial for the host during pathogen infection for modulating both the cholinergic system and immune system [10,14]. However, modulation of CTL expression has been less investigated, except for some reports that have suggested the participation of protein kinase C in the efficient trafficking and functioning of CTLs [10]. Recently, some miRNAs targeting ACh degradation were identified [8], providing new hints for miRNA-mediated modulation of the cholinergic system.

As a class of endogenously encoded small non-coding RNAs, miRNAs could play indispensable roles in the post-transcriptional modulation of gene expression [15,16]. To date, plenty of miRNAs have been identified with modulatory roles in most biological processes, including neural events and immune responses [17–20]. Recently, some miRNAs acting as negotiators between the neuroendocrine system and immune system were also identified and functioned critically in the host response against invading pathogens [21,22]. For instance, it was found that miR-132 could modulate ACh degradation in mammals by targeting acetylcholinesterase and regulate the inflammatory response subsequently during LPS challenge [8]. However, less is known about invertebrate miRNAs and their interactions with the neuroendocrine and immune systems.

As one of the largest phyla in invertebrates, the Mollusca have evolved a primitive neuroendocrine system and immune system which are similar to these found in mammals [23,24]. Among them, the molluscan cholinergic system was also found to modulate the host immune response during pathogen challenge [23,25], making the Mollusca an ideal model to survey the vertebrate neuroendocrine–immune interactions [26]. Lately, dozens of immune-responsive miRNAs have been identified in haemocytes of oyster *Crassostrea gigas* during *Vibrio splendidus* challenge [27], and among them an invertebrate-specific one (cgi-miR-2d) was speculated to modulate the oyster cholinergic system and immune response by targeting CgCTL1 (NCBI accession number: EKC37701). The purposes of this study were therefore to (i) quantify the concentration changes of ACh and choline in oyster haemolymph after the *V. splendidus* challenge, (ii) verify the interaction between cgi-miR-2d and CgCTL1 both *in vitro* and *in vivo*, (iii) investigate their modulation of choline uptake and ACh metabolism and (iv) survey the potential influences on host immune response, hopefully providing new hints for the miRNA-mediated immunomodulatory role in invertebrates.

## 2. Material and methods

### 2.1. Oyster culture, bacterial challenge and sample collection

Oysters *C. gigas* (averaging 150 mm in shell length, 70 mm in width) employed in this study were collected from a local farm in Qingdao, China. A narrow notch was sawn for injection in each oyster shell, adjacent to the adductor muscle. All oysters were then cultured in aerated seawater (about 20°C) for two weeks before use.

A total of 300 oysters from control and bacterial challenge groups were injected individually with 100 µl sterile seawater (SW group) or 100 µl live *V. splendidus* (1 × 10^7^ CFU ml^−1^ in sterile seawater; VS group), respectively [28]. Five oysters were then sampled randomly from each group at 0, 4, 8 and 12 h post-injection to collect the haemocytes from the posterior adductor muscle sinus. After centrifugation at 800*g*, 4°C for 10 min, the haemocytes obtained were lysed by Trizol (Invitrogen, Cat. No. 15596018) and subjected to RNA isolation and subsequently cDNA synthesis. The relative expression level of CgCTL1 was then surveyed by quantitative real-time PCR (qRT-PCR) assay. Another five oysters were also sampled at 12 h to detect haemolymph ACh and haemocyte choline. Each trial was conducted with three replicates.

### 2.2. Knock-down of CgCTL1 *in vivo* by dsRNA-mediated RNA interference

The C-terminus of CgCTL1 coding region (25–618 nt), which was unique in the oyster genome based on BLASTn results, was selected for dsRNA-mediated RNA interference using the method described previously [29]. Briefly, a pair of T7 promoter-linked primers (table 1) was employed to amplify the selected sequence and the PCR products were subsequently subjected to *in vitro* dsRNA transcription using T7 RNA polymerase (Thermo, Cat. No. EP0111). The synthesized dsRNA was then examined for RNA integrity by electrophoresis and quantified using a Nanodrop2000 (Thermo). A cDNA fragment (657 bp) from pEGFP vector (Clontech, Cat. No. 6085-1) was also employed in this study for the synthesis of control dsRNA.

**Table 1.** Primers and RNAs used in this study.

category	primer and RNA name	sequence (5′–3′)
RNAs	cgi-miR-2d	UAUCACAGCCUGCUUGGAUCAGU UGAUCCAAGCAGGCUGUGAUAUU
	miRNA control	UUCUCCGAACGUGUCACGUTT ACGUGACACGUUCGGAGAATT
	cgi-miR-2d inhibitor	ACUGAUCCAAGCAGGCUGUGAUA
	inhibitor control	CAGUACUUUUGUGUAGUACAA
UTR clone primers	CgCTL1_F_UTRclone	TGATGGTGCCAAAGTGACCC
	AP-dT	GGCCACGCGTCGACTAGTACT_17_
	CgCTL1_XhoI	CTCGAGACAGCTCAGCAGGAG
	CgCTL1_NotI	GCGGCCGCATGACTGTACTACTCCTTG
primers for knock-down assay	EGFP-basic-F	CGACGTAAACGGCCACAAGT
	EGFP-basic-R	CTTGTACAGCTCGTCCATGC
	EGFP-T7-F	TAATACGACTCACTATAGGGATC CGACGTAAACGGCCACAAGT
	EGFP-T7-R	TAATACGACTCACTATAGGGATC CTTGTACAGCTCGTCCATGC
	CgCTL1-basic-F	ATTCTGCGGAAGATAAAGCC
	CgCTL1-basic-R	CCAGCCTGTCATTAAATCGTG
	CgCTL1-T7-F	TAATACGACTCACTATAGGGATC ATTCTGCGGAAGATAAAGCC
	CgCTL1-T7-R	TAATACGACTCACTATAGGGATC CCAGCCTGTCATTAAATCGTG
qRT-PCR primers	CgCTL1_F	ACAGCTCAGCAGGAGGAGAGA
	CgCTL1_R	ATGACTGTACTACTCCTTGATGACC
	CgTNF6440_F	TCATTGGAGCACCTGGAGGATAAG
	CgTNF6440_R	AGAACGACACCTGGCTGTAGACG
	CgTNF5109_F	CGCAATGGTCGCTTGGTGGTC
	CgTNF5109_R	CGTAGGGGCGGAAGGTCTCG
	GAPDH_F	AGGTCGGTGTGAACGGATTTG
	GAPDH_R	TGTAGACCATGTAGTTGAGGTCA
	CgEF-F	AGTCACCAAGGCTGCACAGAAAG
	CgEF_R	TCCGACGTATTTCTTTGCGATGT
	cgi-miR-2d	TATCACAGCCTGCTTGGATCAGT
	rRNA_5s	CAAGGATGACACGCAAAT

A total of 45 oysters in the SW group, siEGFP group and siCgCTL1 group were then injected with 100 µl sterile seawater or 100 µg dsRNA of EGFP or CgCTL1 (in 100 µl DEPC water), respectively, for RNA interference. At 24 h post-injection, haemocytes from five oysters in each group were sampled to survey the knock-down efficiency by qRT-PCR and semi-quantitive PCR of CgCTL1.

Another 90 oysters from SW, VS + siEGFP and VS + siCgCTL1 groups were also employed and received the same treatment as mentioned above. At 24 h post-dsRNA injection, oysters in the VS + siEGFP and VS + siCgCTL1 groups were challenged with 100 µl live *V. splendidus* (1 × 10^7^ CFU ml^−1^ in sterile seawater), while oysters in the SW group were re-injected with 100 µl sterile seawater. Haemocytes from five oysters in each group were then sampled 12 h later for qRT-PCR of CgTNFs while haemolymph and haemocytes from the rest of the oysters were also collected for choline/ACh detection.

All trials were conducted with three replicate samples.

### 2.3. miRNA mimics and inhibitor and 3′-UTR luciferase reporter assay

A cgi-miR-2d mimics, identical with endogenous cgi-miR-2d in nucleotide sequence, were synthesized by GenePharma according to the sequence information. A cgi-miR-2d inhibitor, which was in complete base-pair complementation with mature cgi-miR-2d, was also synthesized by GenePharma. Control mimics and inhibitor control which could not target any genes or miRNAs were also provided by GenePharma. All RNAs are listed in table 1.

The 3′-UTR luciferase reporter assay was conducted using methods described previously [30] with some modification. In brief, target genes of cgi-miR-2d were first searched globally using 3′-UTR sequences of oyster genes. The CgCTL1 3′-UTR (119 bp), which contained a putative binding site of cgi-miR-2d from 40 nt to 65 nt, was then obtained using gene-specific primers (table 1) and then inserted into psiCHECK-2 vector (Promega, Cat. No. C8021). HEK293T cells were then employed for the transfection of recombinant vector with cgi-miR-2d mimics or inhibitor using Lipofectamine 2000 reagent (Invitrogen, Cat. No. 11668-027). Cells transfected merely with recombinant vector were employed as blank group. After 24 h, the relative luminescence ratio of cells in each group was detected using a Dual-luciferase Reporter Assay System Kit (Promega, Cat. No. E1910). The relative expression level of CgCTL1 3′-UTR was also surveyed in cells from blank, cgi-miR-2d and miR control groups (human *GAPDH* gene as internal control). The detailed transfection information was listed in the electronic supplementary material, table S1.

Each trial was conducted with three replicates.

### 2.4. Gain-of-function and loss-of-function of cgi-miR-2d *in vivo*

A total of 120 oysters from the VS group, VS_M group and VS_I group were employed for gain- and loss-of-function assays of cgi-miR-2d *in vivo* and were first challenged with 100 µl suspension of live *V. splendidus* (1 × 10^7^ CFU ml^−1^ in sterile seawater). Oysters in VS, VS_M and VS_I groups were then injected with 100 µl DEPC water, 2.5 nmol cgi-miR-2d mimics (in 100 µl DEPC water) and 2.5 nmol cgi-miR-2d inhibitor (in 100 µl DEPC water), respectively. Another 45 oysters, which were injected with 100 µl of sterile seawater and 100 µl of DEPC water, were employed as the SW control group. At 12 h post-injection, haemocytes from five individuals in each group were harvested to survey the expression level of cgi-miR-2d while haemocytes from another five individuals were collected for qRT-PCR of CgCTL1, CgTNF6440 (NCBI accession number: EKC39243) and CgTNF5109 (NCBI accession number: EKC29547). Concentrations of haemocyte cytoplasmic choline, haemolymph ACh and haemolymph choline were also quantified with five oysters in each group.

An additional 45 oysters from SW, VS and VS_I groups were also prepared and sampled likewise to survey haemocyte bacteriostatic activity.

Each trial was conducted with three replicates.

### 2.5. RNA isolation, cDNA synthesis, SYBR Green fluorescent qRT-PCR and semi-quantitive PCR

Total RNA isolation, cDNA synthesis, SYBR Green fluorescent qRT-PCR and semi-quantitive PCR were carried out according to methods described previously [23]. Gene-specific primers used in the above assays are all listed in table 1. Three replicates were applied in all trials. Relative expression levels of related genes were calculated using the 2^−ΔΔCt^ method [31]. Intensity analysis of PCR products from semi-quantitive PCR was conducted with ImageJ software (http://imagej.net/Welcome).

### 2.6. Choline and acetylcholine quantification assay

The concentrations of choline and ACh in oyster haemocytes and haemolymph were measured with a choline/ACh quantification kit (Abnova, Cat. No. ka0840) according to the manual. Briefly, 50 µl haemocyte lysates or haemolymph were added to a mixture containing 44 µl choline assay buffer, 2 µl choline probes and 2 µl enzyme mix and incubated for 30 min at room temperature in darkness. Choline concentration was then determined by colorimetric assay at OD_570_ or by fluorometric assay at Ex/Em = 530/590 nm. ACh concentration was detected likewise except with an addition of 2 µl acetylcholinesterase during the incubation. The haemocyte cytoplasmic choline was further normalized with total cytoplasmic protein.

### 2.7. Bacteriostatic activity assay

Bacteriostatic activity assay against *Staphylococcus aureus* and *Micrococcus luteus* were conducted with methods described previously [32]. Briefly, haemocytes from the SW or VS or VS_I groups were first lysed on ice with homogenizer and centrifuged at 12 000*g*, 4°C for 10 min to obtain the lysates. A total of 50 µl fresh bacteria (1 × 10^5^ CFU ml^−1^ in LB broth) were then employed and incubated with haemocyte lysates (50 µl in PBS, protein concentration at 0.40 mg ml^−1^). After 1 h incubation, these bacteria were cultured at room temperature for 20 h and subjected to OD_600_ detection hourly to indicate growth rate.

### 2.8. Statistical analysis

All data are given as means ± s.d. and were subjected to one-way analysis of variance (one-way ANOVA) followed by a multiple comparison to determine the significant difference. Asterisks or letters above bars indicate *p* *<* 0.05 (* or a, b, c) or *p* *<* 0.01 (**).

## 3. Results

### 3.1. Oyster cholinergic system was vigorously modulated in haemocytes during *Vibrio splendidus* challenge

**Figure 1.**
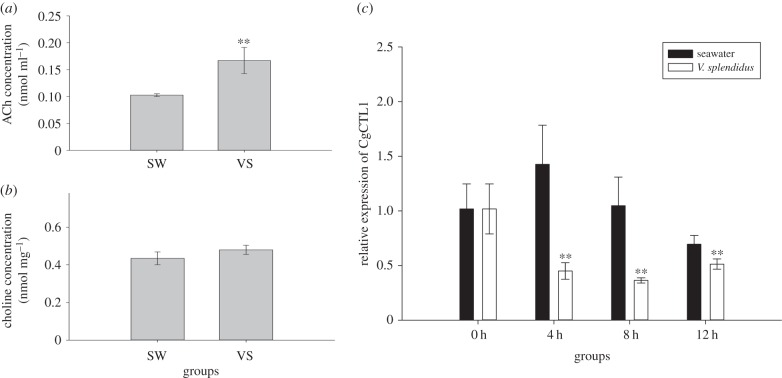
Oyster cholinergic system was vigorously modulated after *Vibrio splendidus* challenge. Concentration of haemolymph ACh (*a*) and haemocyte choline (*b*) were detected at 12 h after seawater injection (SW) or *V. splendidus* challenge (VS). The transcripts of CgCTL1 were found to be vigorously modulated during the early stage of challenge (*c*). Significant differences are marked with asterisks, **p *<* 0.01.

The ACh concentration in oyster haemolymph and the choline concentration in haemocyte cytoplasm were measured at 12 h after the *V. splendidus* challenge. It was found that ACh level increased significantly in oyster haemolymph after the challenge (VS group level 1.62-fold of that in the SW control group, *p* < 0.01) (figure 1*a*) and choline level remained unchanged in haemocyte cytoplasm (figure 1*b*).

The mRNA level of CgCTL1 decreased significantly at 4 h after *V. splendidus* challenge and reached the lowest level at 8 h (0.36-fold of that in the SW control group, *p* < 0.01). At 12 h post-challenge, a significant restoration of CgCTL1 transcripts was observed, increasing to 0.74-fold of that in SW control group (figure 1*c*).

### 3.2. CgCTL1 was responsible for haemocyte choline uptake during challenge

The protein sequence of CgCTL1 retrieved from NCBI (https://www.ncbi.nlm.nih.gov/) was subjected to SMART (http://smart.embl.de/) for structure analysis. It was found that *CgCTL1* encoded a polypeptide of 647 amino acids with three transmembrane domains and one typical ‘choline_transpo’ domain (electronic supplementary material, figure S1*a*). Meanwhile, it also shared high identity (*I*%) and similarity (*S*%) with other CTLs from *Lingula anatine, Rattus norvegicus, Homo sapiens, Mus musculus* and *Xenopus laevis*, etc. (table 2; electronic supplementary material, figure S1*b*).

**Table 2.** The percentage identities (*I*%) and similarities (*S*%) between CTL1 from *Crassostrea gigas* and CTLs in other organisms. *I*%: identity, calculated as the percentage of identical amino acids per position in alignments; *S*%: similarity, calculated as the percentage of identical plus similar residues. *I*% and *S*% were analysed using the Ident and Sim Analysis provided on http://www.bioinformatics.org/sms/.

ID	organism	*I*%	*S*%
XP_013403882	*Lingula anatina*	53.4	65.9
CAB75555	*Rattus norvegicus*	44.2	57.2
CAC82175	*Homo sapiens*	44	57.2
EDL02281	*Mus musculus*	43.7	56.4
ELR46426	*Bos mutus*	43.7	57.1
NP_001085247	*Xenopus laevis*	42.2	56.3
KFW81574	*Manacus vitellinus*	41.9	56.3
ELK17651	*Pteropus alecto*	35.2	45.6
XP_011666027	*Strongylocentrotus purpuratus*	30.4	41.9
XP_012561328	*Hydra vulgaris*	22.7	39.2

**Figure 2.**
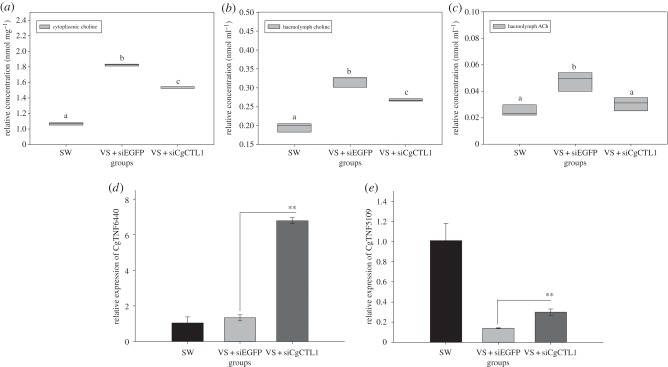
CgCTL1 modulates choline uptake and expression of TNF-like genes in oyster haemocytes. It was found that haemocyte choline (*a*), haemolymph ACh (*b*) and haemolymph choline (*c*) altered significantly during knock-down assay of CgCTL1. (All data are shown in boxplot with lines representing concentration measured. Haemocyte choline was normalized using cytoplasmic protein). Transcripts of CgTNF6440 (*d*) and CgTNF5109 (*e*) also changed markedly after CgCTL1 knock-down. Significant differences are marked by different letters (a, b, c), *p* *<* 0.05; **p *<* 0.01.

To investigate the participation of CgCTL1 in choline import, dsRNA of CgCTL1 was then synthesized and injected into oysters (electronic supplementary material, figure S2*a*). Consequently, a significant decrease of CgCTL1 transcripts was observed at 24 h post-injection in comparison with the SW or siEGFP control group (electronic supplementary material, figure S2*b,c*). The knock-down assay of CgCTL1 was then carried out under the *V. splendidus* challenge. It was observed that cytoplasmic choline level increased significantly in the VS + siEGFP group (in comparison with the SW group) and declined markedly when dsRNA of CgCTL1 was injected (VS + siCgCTL1group, 0.83-fold of VS + siEGFP group, *p* *<* 0.01) (figure 2*a*). Meanwhile, the haemolymph ACh also increased in the VS + siEGFP group and decreased significantly after CgCTL1 knock-down (0.047 nmol ml^−1^ versus 0.030 nmol ml^−1^, *p* *<* 0.01) (figure 2*b*). Comparatively, the haemolymph choline concentration altered to 0.32 nmol ml^−1^ in the VS + siEGFP group and descreased to 0.26 nmol ml^−1^ in the VS + siCgCTL1 group (figure 2*c*).

The expression of two TNF-like genes (CgTNF6440 and CgTNF5109) was altered likewise in oyster haemocytes after CgCTL1 knock-down. Specifically, CgTNF6440 (CGI_10006440, EKC39243) transcripts remained unchanged in the VS + siEGFP group and increased significantly in the VS + siCgCTL1 group (5.78-fold of that in VS + siEGFP group, *p* *<* 0.01) (figure 2*d*). CgTNF5109 (CGI_10005109, EKC29547) transcripts decreased significantly in the VS + siEGFP group (0.14-fold of SW group, *p* *<* 0.01) yet recovered markedly in the VS + siCgCTL1 group (0.28-fold of that in SW group, *p* *<* 0.01) (figure 2*e*).

### 3.3. CgCTL1 could be modulated by cgi-miR-2d both *in vitro* and *in vivo*

**Figure 3.**
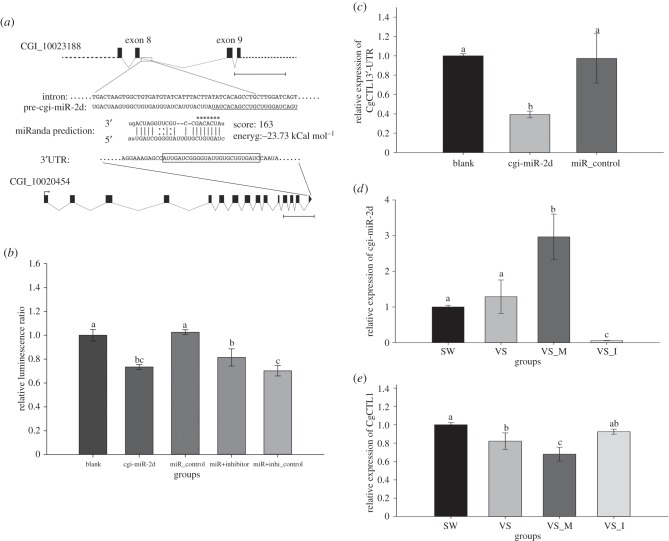
Interactions of cgi-miR-2d and CgCTL1 *in vitro* and *in vivo*. (*a*) CgCTL1 was predicted as a putative target of cgi-miR-2d by miRanda software with a binding site at its 3′-UTR. (*b*) Relative luminescence ratio was detected in HEK293T cells transfected with recombinant vector and cgi-miR-2d mimics (cgi-miR-2d) or miRNA control (miR_control) or cgi-miR-2d + inhibitor (miR + inhibitor) or cgi-miR-2d + inhibitor control (miR + inhi_control). (*c*) Expression levels of CgCTL1 3′-UTR were also surveyed simultaneously in cells from blank, cgi-miR-2d and miR_control groups. (*d*) Changes in cgi-miR-2d transcript levels were investigated by qRT-PCR after its gain- and loss-of-function assay *in vivo*. (*e*) CgCTL1 transcript levels altered markedly in gain- and loss-of-function of cgi-miR-2d *in vivo*. Significant differences were marked by different letters (a, b, c), *p* *<* 0.05.

The 3′-UTR of CgCTL1, which contains a putative binding site of cgi-miR-2d (figure 3*a*; electronic supplementary material, figure S3), was cloned and subjected to luciferase reporter assay to survey its interaction with cgi-miR-2d *in vitro*. As a result, a significant decrease of the relative luminescence ratio was observed in HEK293-T cells transfected with cgi-miR-2d mimics (0.73-fold of that in blank group, *p* *<* 0.01), while the luminescence recovered to 0.81-fold that of the blank group after co-transfection of cgi-miR-2d inhibitor (miR + inhibitor group) (figure 3*b*). The transfection of inhibitor control, however, failed to rescue the decrease resulting from cgi-miR-2d, which was 0.70-fold of that in the blank group (miR + inhi_control group, *p* *<* 0.01) (figure 3*b*).

At the same time, a significant decrease of CgCTL1 3′-UTR was also observed in the cgi-miR-2d transfection group in comparison with either blank or miR_control group (0.61-fold of that in the blank group, *p* *<* 0.01) (figure 3*c*).

The CgCTL1 transcripts also were markedly affected by gain- and loss-of-function of cgi-miR-2d *in vivo*. In detail, CgCTL1 transcripts decreased significantly in the VS_M group (0.82-fold of VS group, *p* *<* 0.05) (figure 3*e*) when cgi-miR-2d transcripts were overexpressed (2.30-fold of that in VS group, *p* < 0.01) (figure 3*d*). When cgi-miR-2d transcripts were inhibited by its inhibitor (VS_I group, 0.05-fold of that in VS group, *p* < 0.01) (figure 3*d*), CgCTL1 transcripts increased to a moderate level in between that of the SW and VS groups (1.04-fold of VS group, *p* < 0.05) (figure 3*e*).

### 3.4. Modulation of choline and acetylcholine concentration by cgi-miR-2d

**Figure 4.**
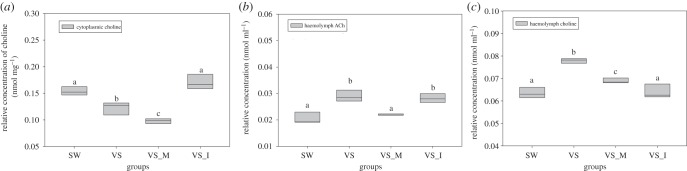
Modulations of choline and Ach concentrations by cgi-miR-2d. Haemocyte choline (*a*), haemolymph ACh (*b*) and haemolymph choline (*c*) were found to be changed significantly in gain- and loss-of-function assay of cgi-miR-2d. (All data are shown in boxplot with lines representing concentration measured. Haemocyte choline was normalized using cytoplasmic protein.) Significant differences are marked by different letters (a, b, c), *p* *<* 0.05.

Consistent with changes in CgCTL1 expression, choline concentration in oyster haemocytes also decreased significantly in the VS_M group compared with VS group (0.10 nmol mg^−1^, *p* < 0.01) (figure 4*a*). When cgi-miR-2d was inhibited, however, cytoplasmic choline increased significantly to 0.17 nmol mg^−1^ in the VS_I group (*p* < 0.01) (figure 4*a*).

Significant changes in the haemolymph ACh and choline were also observed in the gain- and loss-of-function assay of cgi-miR-2d. It was found that haemolymph ACh increased to 0.029 nmol ml^−1^ after *V. splendidus* infection (compared with 0.020 nmol ml^−1^ in SW group, *p* < 0.01) and decreased to 0.022 nmol ml^−1^ when cgi-miR-2d was overexpressed (*p* < 0.01) (figure 4*b*). No significant changes of the ACh concentration were observed in oysters receiving co-injection of bacteria and cgi-miR-2d inhibitor in comparison with the VS group (0.028 nmol ml^−1^) (figure 4*b*).

The haemolymph choline concentration also peaked at 12 h post-bacterial challenge (0.078 nmol ml^−1^, *p* < 0.01) and decreased to 0.069 nmol ml^−1^ when cgi-miR-2d was co-injected with bacteria (figure 4*c*). Meanwhile, a robust reduction of haemolymph choline was observed in the VS_I group, in which cgi-miR-2d was repressed (0.063 nmol ml^−1^) (figure 4*c*).

### 3.5. Cgi-miR-2d promoted expression of tumour necrosis factor-like genes and haemocyte anti-bacterial activity

**Figure 5.**
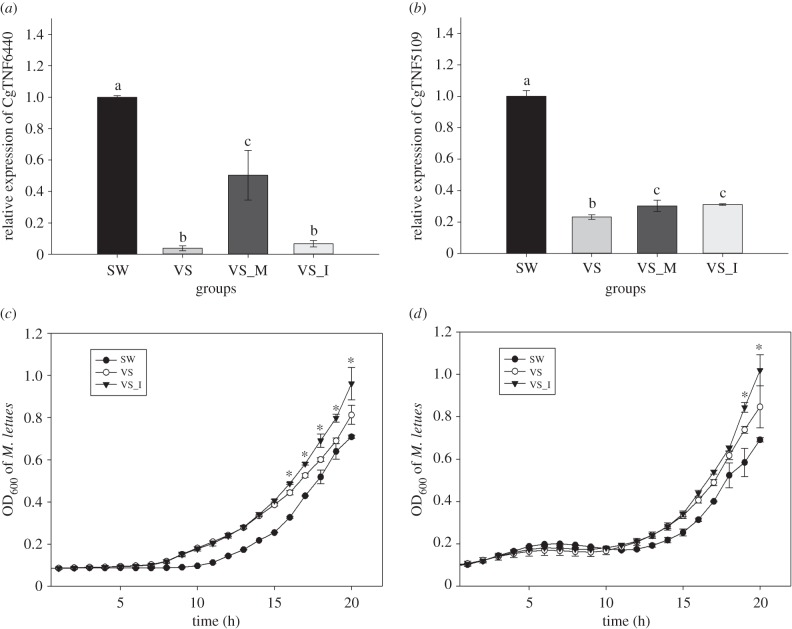
Cgi-miR-2d modulated expression of oyster TNF-like genes and the haemocyte bacteriostatic activity. Transcripts of CgTNF6440 (*a*) and CgTNF5109 (*b*), two TNF-like genes in oyster haemocytes, were surveyed in gain- and loss-of-function assay of cgi-miR-2d by qRT-PCR. Bacteriostatic activity against *Staphylococcus aureus* (*c*) and *Micrococcus luteus* (*d*) was also detected using haemocyte lysates when cgi-miR-2d was inhibited. Significant differences are marked by different letters (a, b, c) or asterisk, *p* *<* 0.05.

Expression levels of CgTNF6440 and CgTNF5109 were subsequently surveyed. As a result, the mRNA levels of these two TNF-like genes decreased significantly after bacterial challenge and were restored after cgi-miR-2d overexpression (*p* *<* 0.01) (figure 5*a,b*). Comparatively, when cgi-miR-2d was repressed during *V. splendidus* challenge, CgTNF6440 transcripts were maintained at a significantly lower level than that in either SW or VS_M groups (*p* *<* 0.01) (figure 5*a*), whereas CgTNF5109 transcripts increased to a level comparable with that in VS_M group (*p* *<* 0.01) (figure 5*b*).

The bacteriostatic activity of haemocyte lysates was also hampered after the inhibition of cgi-miR-2d. In detail, the growth rate of *S. aureus* and *M. luteus* was significantly promoted in the VS group compared with that in the SW control group and increased further in the VS_I group where cgi-miR-2d was inhibited (figure 5*c,d*).

## 4. Discussion

The interaction between the cholinergic system and immune system plays important roles in the host's defence against invading pathogens [33]. It has been suggested that the cholinergic system could be activated at a late stage of infection with anti-inflammatory roles [5]. At the same time, some miRNAs might contribute crucially to the continuation of the anti-inflammatory signalling by repressing ACh degradation [8]. However, less is known about the early stage of infection when the cholinergic system is repressed. Recently, a primitive cholinergic system was characterized in Mollusca [20,21,25], providing opportunities for further study on the interaction between the cholinergic system and immune system. Here, an invertebrate-specific miRNA in oyster *C. gigas* was proved to suppress the synthesis and release of ACh at an early stage of pathogen challenge by targeting CgCTL1, and led subsequently to the promotion of host immune response.

As reported in scallops, the neurotransmitter ACh could be accumulated robustly in haemolymph after LPS stimulation and modulate immune responses simultaneously [23]. In this study, an increase of haemolymph ACh was also observed in oysters at 12 h post-*V. splendidus* challenge (figures 1*a*, 2*b* and 4*b*), while the cytoplasmic choline concentration remained at a relatively stable level at the same time (figures 1*a*, 2*a* and 4*a*), demonstrating the activation of the oyster cholinergic system during pathogen challenges. Meanwhile, CgCTL1, which shares conserved protein sequence and structure with its mammalian homologues (electronic supplementary material, figure S1; table 2), was found to be downregulated sharply at the early stage of infection, yet recovered at 12 h post-bacteria injection (figure 1*c*), indicating its participation in ACh metabolism during challenge. Knock-down assay of CgCTL1 was then conducted *in vivo* during *V. splendidus* challenge to verify its function. Subsequently, a significant decrease of haemocyte choline was observed when CgCTL1 was repressed (figure 2*a,b*; electronic supplementary material, figure S2), demonstrating the repression of choline uptake. Meanwhile, the haemolymph ACh was also downregulated significantly, suggesting CgCTL1's participation in ACh synthesis and release. Together, it was suggested that CgCTL1 could contribute indispensably to the modulation of the oyster cholinergic system after pathogen stimulation, while the modulatory mechanisms on CgCTL1 remained largely unknown.

As a crucial class of gene regulators at post-transcription level, most miRNAs could bind to the 3′-UTR region of target genes and subsequently lead to their degradation or translation repression [34]. In previous studies, some immune-responsive miRNAs were identified in oysters [27] and were reassessed here for their involvement in expressional modulation of CgCTL1. As a result, the miRNA cgi-miR-2d increased significantly during *V. splendidus* challenge, and was predicted to target CgCTL1 via the 3′-UTR region [27]. Moreover, an opposite expression pattern of cgi-miR-2d was observed during *V. splendidus* challenge in comparison with CgCTL1 [35], indicating their interaction *in vivo*. CgCTL1 3′-UTR luciferase reporter assay was then conducted *in vitro* to verify the interaction. As a result, both the relative luminescence ratio and CgCTL1 3'-UTR transcripts were found decreased after cgi-miR-2d transfection (figure 3*b,c*). In addition, CgCTL1 transcripts were also significantly downregulated in gain-of-function of cgi-miR-2d *in vivo* during bacterial challenge (figure 3*d,e*). These results collectively confirmed the interaction between cgi-miR-2d and CgCTL1 in oyster haemocytes, which was proposed to be triggered rapidly at the early stage of infection.

Though thousands of miRNAs have been identified to date, few of them have been found to interact with both immune system and neuroendocrine systems, especially the cholinergic system [21]. In mammals, miR-132 was proved to modulate ACh degradation by targeting acetylcholinesterase [8]. Given the function of CgCTLs in choline transport and ACh synthesis (figure 2) [36], cgi-miR-2d was speculated to regulate both choline uptake and ACh synthesis during challenge. In support of this speculation, cytoplasmic choline level decreased markedly in oysters injected with cgi-miR-2d, and returned to the basal level when cgi-miR-2d was inhibited (figure 4*a*). Meanwhile, the significant increase of haemolymph ACh induced by pathogen stimulation was also reversed by cgi-miR-2d (figure 4*b*). Generally, haemolymph choline could be vigorously affected by either haemocyte choline uptake or haemolymph ACh degradation [1,25], making it another hallmark of interaction between cgi-miR-2d and CgCTL1. In this study, haemolymph choline increased significantly in the VS group accompanied with the increase of haemolymph ACh and the decrease of choline uptake (figures 2*c* and 4*c*). In addition, the haemolymph choline decreased markedly after either cgi-miR-2d overexpression or CgCTL1 knock-down owing to the repression in both choline uptake and the synthesis/release of ACh (figure 4*c*). When cgi-miR-2d-mediated repression of both choline uptake and ACh synthesis/release was inhibited, haemolymph choline was found decreased more sharply (figure 4*c*). Together, these results demonstrated the modulation of both choline uptake and ACh synthesis/release by cgi-miR-2d, which would result crucially in the temporal changes of the oyster cholinergic system during bacterial challenge.

**Figure 6.**
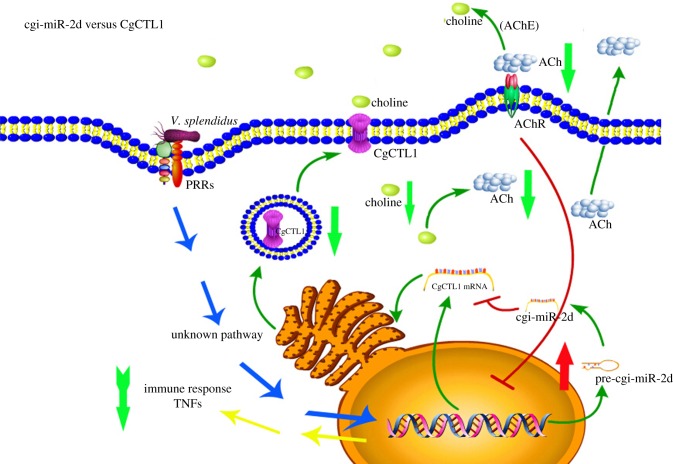
Proposed role of cgi-miR-2d in immune response against *Vibrio splendidus* challenge by interacting with the oyster cholinergic system. cgi-miR-2d is proposed to suppress choline uptake into oyster haemocytes at the early stage of infection by targeting CgCTL1, resulting in the subsequent repression on ACh synthesis and release. Immune responses such as expression of TNF-like genes and haemocyte bacteriostatic activity can be modulated simultaneously for fast elimination of invaded pathogens. At the late stage of infection, expression of cgi-miR-2d is repressed while expression of CgCTL1 is upregulated, leading to repressed immune responses of haemocytes.

As reported, the expression of multiple immune-related genes, including some cytokines and antibacterial peptides [6,7,25], could be vigorously modulated by ACh during pathogen challenge [9]. Among these genes, TNFs are of importance as they can elicit various immune responses as pro-inflammatory cytokines, including humoral and cellular ones [37]. Though less characterized, multiple members of the TNF superfamily have been found in oysters [38]. Some of them were also found rapidly induced during pathogen challenge, indicating their roles in oyster immune response [39]. Given that haemolymph ACh could be downregulated by cgi-miR-2d, the immune response of oyster haemocytes was also suggested to be shaped simultaneously. In this study, the expression level of two TNF-like genes (CgTNF6440 and CgTNF5109) were surveyed in oyster haemocytes during gain- and loss-of-function assay of cgi-miR-2d. It turned out that the transcripts of the above TNF-like genes were significantly downregulated at 12 h post-challenge when haemolymph ACh increased. In contrast, these transcripts increased remarkably when ACh was repressed by cgi-miR-2d overexpression, which was consistent with results in CgCTL1 knock-down assay, confirming our speculation (figures 2*d,e* and 5*a,b*). In addition to the expression of pro-inflammatory cytokines, the bacteriostatic activity of haemocyte lysates could also be indicative of immune response [40,41]. As demonstrated in Mollusca, both ACh and TNFs could modulate expression of antibacterial genes [23,39]. Considering the interaction between cgi-miR-2d and ACh/TNF, it was therefore inferred that inhibition of cgi-miR-2d during the infection could lead to the significant inhibition of haemocyte bacteriostatic activity. In support of our hypothesis, a significant promotion of haemolymph bacteriostatic activity against *V. splendidus* was observed in oysters injected with oyster TNF-like protein [39]. As demonstrated in other reports, *S. aureus* and *M. luteus* have been considered as two model bacteria in investigating immune responses of marine invertebrates [40,42,43]. Here, the bacteriostatic activities of haemocyte lysates against *S. aureus* and *M. luteus* also decreased significantly when cgi-miR-2d was repressed (figure 5*c,d*). Besides, previous reports have also demonstrated the modulation of oyster miRNAs by ACh, which could elicit a more complex immunomodulatory network in haemocytes [22]. It is therefore suggested that the interaction between cgi-miR-2d and CgCTL1 could contribute crucially to the sophisticated immunomodulation of oyster haemocytes during the early stage of infection (figure 6). To date, more than 28 000 miRNAs have been identified across plants and animals, among which miR-2d was found expressed exclusively in invertebrates [44]. This study on the interaction between an invertebrate-specific miRNA and the ancient cholinergic system, therefore, can also shed new light in further understanding the onset of interaction between the neuroendocrine system and immune system, as well as the survival strategy of oysters thriving in harsh intertidal environments.

## Supplementary Material

Supplement file

## Data accessibility

DNA and protein sequences are obtained from GenBank under accession numbers EKC37701, EKC39243, EKC29547, XP_013403882, CAB75555, CAC82175, EDL02281, ELR46426, NP_001085247, KFW81574, ELK17651, XP_011666027 and XP_012561328. The phylogenetic data employed in the study were submitted to TreeBASE with accession number S19473. Final DNA sequence of 3′-UTR of CgCTL1 has been uploaded as the electronic supplementary material.
