# Supplementary material for: An invertebrate-specific miRNA targeted the ancient cholinergic neuroendocrine system of oyster
Source: Open Biol. 2016 Aug 3;6(8):160059. doi: 10.1098/rsob.160059 (PMC5008008; doi:10.1098/rsob.160059)
Supplement: Supplement file [file rsob160059supp1.pdf]

An invertebrate-specific miRNA targeted the ancient cholinergic neuroendocrine system of oyster

Hao Chen[1,3], Zhi Zhou[1], Lingling Wang[2*], Hao Wang[1], Rui Liu [1], Huan Zhang[1], Linsheng Song[1,2*]

[1] Key Laboratory of Experimental Marine Biology, Institute of Oceanology, Chinese Academy of Sciences, Qingdao 266071, China

[2] Key Laboratory of Mariculture & Stock enhancement in North China's Sea, Ministry of Agriculture, Dalian Ocean University, Dalian 116023, China

[3] University of Chinese Academy of Sciences, Beijing 100049, China

*Correspondence to:

Dr. Lingling Wang,

Dalian Ocean University, 52 Heishijiao Street, Dalian 116023, China.

Tel: + 86 411 84763003

Email address: wanglingling@dlou.edu.cn;

Dr. Linsheng Song

Dalian Ocean University, 52 Heishijiao Street, Dalian 116023, China.

Tel: + 86 411 84763173

Email address: lshsong@dlou.edu.cn

Supplementary Figure S1. Protein structure and phylogenic analysis of CgCTL1.

A

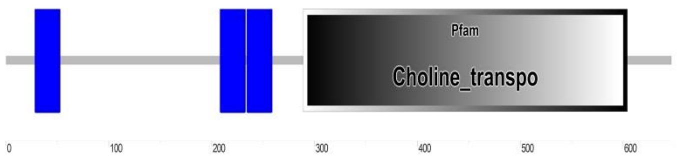

B

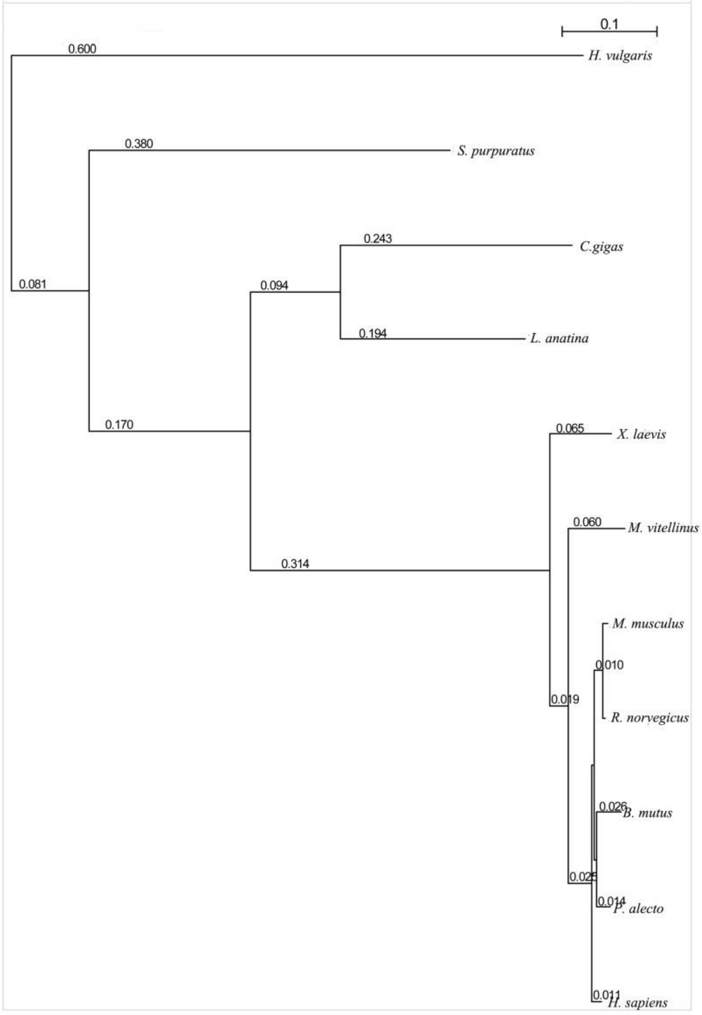

(A) Protein structure of CgCTL1 was predicted by SMART (http://smart.embl.de/). (B) Homologues of CTL1 from *Hydra vulgaris*, *Strongylocentrotus purpuratus*, *Crassostrea gigas*, *Lingula anatine*, *Xenopus laevis*, *Manacus vitellinus*, *Mus musculus*, *Rattus norvegicus*, *Bos mutus*, *Pteropus alecto* and *Homo sapiens* were subjected for phylogenic analysis using Seaview based on neighbour-joining algorithm (1000 bootstrap).

Supplementary Figure S2. CgCTL1 knock-down *in vivo*.

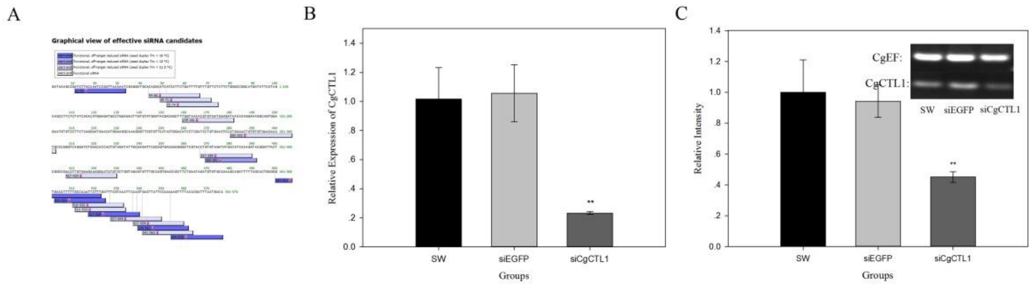

(A) DsRNA of CgCTL1 was designed by siDirect (http://sidirect2.rnai.jp/). (B) Relative expression of CgCLT1 was surveyed by qRT-PCR at 24 h post dsRNA injection. (C) semi-quantitive PCR of CgCTL1 was also conducted after the knock-down and PCR products were subjected to intensity analysis by ImageJ software (http://imagej.net/Welcome).

Supplementary Figure S3. DNA sequence of CgCTL1 3'-UTR.

```
  1 ACAGCTCAGC AGGAGGAGAG ATGGTGAAAG GAAAGAGCCA TTGATCGGGG TATTGTGCTG
 61 TGATCCAATA CTCACAATGT GTTGGTCATC AAGGAGTAGT ACAGTCATCT AGATTTAACA
121 AAAAAAAAAA AAAAAAA
```

Supplementary Table S1. Group and transfection information of the 3'UTR luciferase reporter assay.

Group	Blank	cgi-miR-2d	miR_control	miR+ inhibitor	miR+ inhi_control
Plasmid	100ng	100ng	100ng	100ng	100ng
Cgi-miR-2d		5 pmol		5 pmol	5 pmol
MiRNA Control			5 pmol		
Cgi-miR-2d Inhibitor				5 pmol	
Inhibitor Control					5 pmol
